# Contrasting Cardiopulmonary Responses to Incremental Exercise in Patients with Schistosomiasis-Associated and Idiopathic Pulmonary Arterial Hypertension with Similar Resting Hemodynamic Impairment

**DOI:** 10.1371/journal.pone.0087699

**Published:** 2014-02-03

**Authors:** Fabricio Martins Valois, Luiz Eduardo Nery, Roberta Pulcheri Ramos, Eloara Vieira Machado Ferreira, Celia Camelo Silva, Jose Alberto Neder, Jaquelina Sonoe Ota-Arakaki

**Affiliations:** 1 Division of Respiratory Diseases, Department of Medicine, Federal University of Sao Paulo (UNIFESP), Sao Paulo, Brazil; 2 Division of Cardiology, Department of Medicine, Federal University of Sao Paulo (UNIFESP), Sao Paulo, Brazil; 3 Division of Respiratory and Critical Care Medicine, Department of Medicine, Queen’s University, Kingston, Canada; Vanderbilt University Medical Center, United States of America

## Abstract

It has been reported that schistosomiasis-associated PAH (Sch-PAH) has a more benign clinical course compared with idiopathic PAH (IPAH). We therefore hypothesized that Sch-PAH subjects would present with less impaired cardiopulmonary and metabolic responses to exercise than IPAH patients, even with similar resting pulmonary hemodynamic abnormalities. The aim of this study was to contrast physiologic responses to incremental exercise on cycle ergometer between subjects with Sch-PAH and IPAH. We performed incremental cardiopulmonary exercise tests (CPET) in subjects newly diagnosed with IPAH (n = 9) and Sch-PAH (n = 8), within 1 month of the hemodynamic study and before the initiation of specific therapy for PAH. There were no significant between-group differences in cardiac index, pulmonary vascular resistance or mean pulmonary artery pressure. However, mean peak oxygen uptake (VO_2_) was greater in Sch-PAH than IPAH patients (75.5±21.4 vs 54.1±16.1% predicted, p = 0.016), as well as the ratio of increase in VO_2_ to work rate (8.2±1.0 vs 6.8±1.8 mL/min/W, p = 0.03). Additionally, the slope of the ventilatory response as a function of CO_2_ output was lower in Sch-PAH (40.3±3.9 vs 55.6±19.8; p = 0.04), and the heart rate response for a given change in VO_2_ was also diminished in Sch-PAH compared to IPAH (80.1±20.6 vs 123.0±39.2 beats/L/min; p = 0.02). In conclusion, Sch-PAH patients had less impaired physiological responses to exercise than IPAH subjects with similar resting hemodynamic dysfunction. Our data suggest a more preserved cardiopulmonary response to exercise in Sch-PAH which might be related to its better clinical course compared to IPAH.

## Introduction

Schistosomiasis is potentially the most common cause of pulmonary arterial hypertension (PAH) worldwide. [1], [2] Although 80 years have passed since the first description of cardiopulmonary involvement caused by schistosoma species, this association has not been well studied. [3] Thus, little is known about the pathophysiology, natural history and therapeutic response of schistosomiasis-associated PAH (Sch-PAH).

A recent study demonstrated that naïve Sch-PAH patients had similar survival rates compared to idiopathic PAH (IPAH) subjects treated with vasodilators. In an additional analysis, the authors suggested that Sch-PAH patients have a more benign clinical course than those with IPAH. [4] The mechanisms related to this finding were not described, but it might be related to a slower progression of arteriopathy in Sch-PAH.

Clinicians evaluate functional impairment in PAH patients using the New York Heart Association functional class assessment (NYHA-FC), six-minute walking distance (6MWD) and/or cardiopulmonary exercise testing (CPET). [5], [6] However, only CPET is able to properly identify the physiological mechanisms linked to functional impairment, including ventilatory limitation, circulatory dysfunction or impaired oxygen utilization in peripheral muscles. [7], [8] In fact, CPET has emerged as a surrogate method to evaluate functional impairment in many diseases, and there is a robust body of evidence addressing the diagnostic and prognostic usefulness of CPET in PAH patients.[7]–[13] Moreover, several abnormal CPET findings have been linked to PAH pathophysiology and disease severity. Compared to healthy subjects, patients with PAH show a reduced aerobic capacity, a high slope of minute-ventilation change as a function of CO_2_ production [(ΔVE/ΔVCO_2_), a marker of ventilatory inefficiency], an abnormally diminished end-tidal partial pressure of CO_2_ (PETCO_2_) at the anaerobic threshold and a steeper increase in heart rate as a function of the metabolic demand (ΔHR/ΔVO_2_). These abnormalities have been ascribed to the inability of the right ventricle to increase cardiac output through the pulmonary vascular bed, inefficient gas exchange due to ventilation-perfusion mismatching, and/or abnormalities in the control of ventilation. [7], [8] In this context, it is conceivable that patients with Sch-PAH would present with less severe hemodynamic abnormalities during incremental exercise compared with those with IPAH. To the best of the authors’ knowledge, this assertion has not been previously investigated. So, the aim of this study was to contrast the physiological responses during CPET of Sch-PAH and IPAH patients. We hypothesized that Sch-PAH subjects would have better functional performance compared to IPAH patients as indicated by higher maximal aerobic capacity and less impaired ventilatory, cardiovascular and gas exchange responses to exercise.

## Materials and Methods

### Subjects

This was a cross-sectional study in which incremental CPET was performed in subjects diagnosed with IPAH and Sch-PAH between 2007 and 2010. The test was performed within 1 month of the hemodynamic study and before the initiation of specific therapy for PAH.

PAH was defined as a mean pulmonary artery pressure (mPAP) ≥25 mmHg with a pulmonary capillary wedge pressure (PCWP) ≤15 mmHg. [5], [6] Patients were classified as having IPAH when a routine investigation did not find any cause for PAH. Sch-PAH was characterized by the presence of PAH associated with liver ultrasonographic findings suggestive of hepatoesplenic schistosomiasis (periportal fibrosis and/or left lobe enlargement) and at least one of the following: exposure to a region endemic for schistosomiasis, previous treatment for schistosomiasis or the presence of *Schistosoma mansoni* eggs in a stool examination or rectal biopsy. [1], [4].

Subjects were excluded from study if they had evidence of any of the following: right-to-left intracardiac shunt, resting hypoxemia, hospitalization in the last month, or an orthopedic limitation to cycling. None of the patients had ever been admitted to a physical rehabilitation program.

### Measurements

At the baseline evaluation, we obtained a medical history that included an NYHA-FC assessment, transthoracic echocardiogram, spirometry, single breath carbon monoxide diffusing capacity (DL_CO_), resting arterial blood gases and 6MWD from all patients. [14].

### Hemodynamic Evaluation

A right-sided heart catheterization was performed using standard techniques. Cardiac output (CO) was determined by the Fick method, with arterial and venous line, and estimated VO_2_ (125x body surface area). [15]–[17] Acute vasodilator responsiveness was evaluated using inhaled nitric oxide for 10 minutes, with a positive response defined as a fall in the mPAP of 10 mmHg or more (to a value ≤40 mmHg) without a decrease in CO. [5], [6].

### Cardiopulmonary Exercise Testing (CPET)

Patients performed a symptom-limited ramp-incremental CPET on an electronically braked cycle ergometer (Corival 400, Lode, The Netherlands) at 60 rpm. O_2_ uptake (VO_2,_ L/min), CO_2_ output (VCO_2_, L/min), minute ventilation (VE, L/min), the respiratory exchange ratio (RER, VCO_2_/VO_2_) and the end-tidal partial pressures for CO_2_ (PETCO_2,_ mmHg) and O_2_ (PETO_2,_ mmHg) were measured using a computer-based system, with breath-by-breath analysis (CardiO_2_ System™, Medical Graphics, St. Paul, MN) and were recorded as mean of 15 s. Heart rate (HR, bpm) was recorded from the R-R distance in 12-lead ECG tracing with on-line calculation of O_2_ pulse (VO_2_/HR, ml/beat) and also sampled as mean of 15 s. The peak VO_2_ was compared to Brazilian standards. [18].

The anaerobic threshold (AT) was identified using the modified V-slope and the ventilatory method as previously described. The ΔHR/ΔVO_2_, ΔVE/ΔVCO_2_ and the slope of VO_2_ as a function of work rate (ΔVO_2/_ΔWR) during the incremental phase were calculated from the start of increases in VO_2_ to the respiratory compensation point, according to standard procedures. [7],[19].

Cuff blood pressure and pulse oximetry were monitored and recorded. At the end of the test, subjects were asked to rate their “dyspnea” and “leg fatigue” using the 0–10 Borg’s category-ratio scale. [20].

### Ethics Statement

The study protocol was approved by the Institutional Medical Ethics Committee: Hospital São Paulo – UNIFESP (n° 04878612.4.0000.5505) and written informed consent was obtained from all subjects.

### Statistical Analysis

Statistical analysis was performed using the SPSS 19 statistical package (SPSS, Inc., Chicago, IL). All continuous variables had normal distribution as stated by the Kolmogorov-Smirnov test. Therefore, an unpaired t-test was used for the comparison of resting hemodynamics and exercise responses. We used ANOVA with Bonferroni correction for multiple comparisons. Pearson’s product moment correlation or Spearman’s rank correlation was used according to assess the level of correlation between variables. A p value of less than 0.05 was considered statistically significant.

## Results

### Patient Population

In our cohort, there were 9 subjects with IPAH and 8 subjects with Sch-PAH who met the study criteria and were able to enter the study. At the baseline evaluation, the demographic characteristics of the IPAH and Sch-PAH groups were similar (Table 1). Most patients were in functional class II or III, and there were no differences in 6MWD between the 2 groups. None of the patients with Sch-PAH had anemia or abnormal liver function tests.

**Table 1:** Baseline patient characteristics.

	Sch-PAH	IPAH	p value
Age, years	51±8	44±19	0.36
Gender, n			0.07
Male	4	2	
Female	4	7	
BMI, kg/m^2^	24.6±4	23.7±2	0.62
NYHA functional class			0.95
II	3	3	
III	3	4	
IV	2	2	
FVC, %	81.5±13	91.8±25	0.32
FEV_1_, %	78.4±13	88.8±22	0.29
FEV_1_/FVC	0.79±0.1	0.83±0.1	0.23
DL_CO_, %	63±11	61±10	0.72
PaCO_2_, mmHg	33±3	33±6	0.93
PaO_2_, mmHg	75±10	78±10	0.44
Six-minute-walk distance, m	474±62	462±78	0.40

*Definition of Abbreviations: BMI, body mass index; NYHA, New York Heart Association; FVC, forced vital capacity; FEV, forced expiratory volume; DL_CO_, carbon monoxide diffusing capacity; PaCO_2_, arterial carbon dioxide partial pressure; PaO_2_, arterial oxygen partial pressure.*

### Resting Pulmonary Hemodynamics

Hemodynamic parameters are summarized in Table 2. There were no significant differences in mean pulmonary pressure (mPAP), pulmonary vascular resistance (PVR), or cardiac index (CI) between groups. No patient displayed positive acute vasodilator response to inhaled nitric oxide.

**Table 2:** Hemodynamic characteristics.

Parameter	Sch-PAH	IPAH	p value
Mean pulmonary artery pressure, mmHg	57.5±18.5	63.8±17.1	0.47
Pulmonary capillary wedge pressure, mmHg	12.6±2.8	9.9±3.3	0.10
Right atrial pressure, mmHg	11.9±5	9.7±2	0.26
Pulmonary vascular resistance, IU	12.3±5.6	14.8±8.1	0.46
Cardiac output, L/min	4.00±0.6	4.09±1.2	0.84
Cardiac index, L/min/m^2^	2.37±0.5	2.49±0.7	0.69
Acute vasodilator response, n	0	0	

### Physiological Responses to Exercise

**Figure 1:**
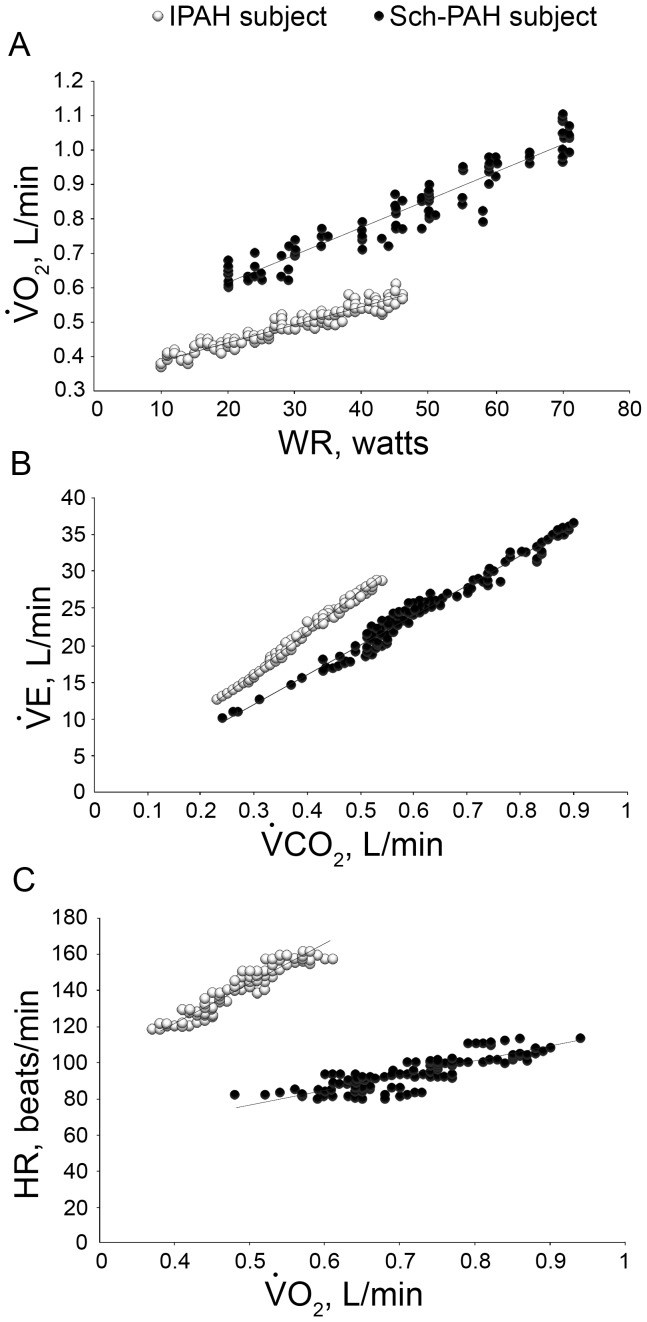
Contrasting exercise responses in two subjects with similar resting hemodynamic impairment (CI 2.24 L/min/m^2^ for Sch-PAH subject, and CI 2.21 L/min/m^2^ for IPAH patient). *Upper panel*: the ratio of increase in VO_2_ to work rate (ΔVO_2_/ΔWR); *middle panel:* slope of VE-VCO_2_; *lower panel*: increases in heart rate as a function of VO_2_.

All subjects exhibited diminished aerobic capacity, with the test being interrupted by symptoms of breathlessness in 66% of IPAH subjects and leg effort in 75% of Sch-PAH subjects. The measurements of CPET variables in each group are presented in Table 3 and the typical physiological responses of Sch-PAH and IPAH patients to selected variables obtained during the CPET are presented in Figure 1.

**Table 3:** Cardiopulmonary exercise testing parameters.

	Sch-PAH	IPAH	p value
WR, watts	84±29	52±17	0.006
Metabolic parameters			
VO_2_ peak, % predicted	75.5±21.4	54.0±16.1	0.016
VO_2_ peak, mL/kg/^/^min	17.5±5.3	12.6±3.2	0.016
VO_2_ at AT, % predicted	51.3±9.3	40.3±13.0	0.040
ΔVO_2_/ΔWR, mL/min^/^W	8.2±1.0	6.8±1.8	0.030
Ventilatory parameters			
VT at AT, L	1.1±0.3	0.9±0.2	0.090
VE at AT, L/min	25.6±6.2	22.6±5.2	0.300
VE/MMV peak	0.64±0.17	0.53±0.17	0.220
VE/VO_2_ at AT	33.6±6.0	42.3±9.4	0.056
VE/VCO_2_ at AT	38.8±5.5	46.6±10.8	0.055
ΔVE/ΔVCO_2_	40.3±3.9	55.6±19.8	0.020
Cardiovascular parameters			
HR at peak, % predicted	85.6±9.7	79.0±10.0	0.200
O_2_ Pulse, % predicted	87.5±20.9	65.5±16.4	0.015
ΔHR/ΔVO_2_, beat/L^/^min	80.1±20.6	123.0±39.2	0.020
Gas exchange parameters			
PETCO_2_ at AT, mmHg	31.0±3.8	28.5±6.1	0.18
PETO_2_ at AT, mmHg	102.1±4.2	104.1±6.8	0.50
O_2_ sat at rest, %	96±2	96±1	0.64
O_2_ sat at peak exercise, %	93±4	94±4	0.58
Subjective parameters			
Borg - Dyspnea at peak	7±2	6±2	0.52
Borg - Leg effort at peak	7±2	5±2	0.09

*Definition of Abbreviations: WR, work rate; VO_2_, oxygen uptake; VE, minute ventilation; AT, anaerobic threshold; VT, tidal volume; MMV, maximal minute ventilation; VCO_2_, carbon dioxide uptake; HR, heart rate; PETCO_2_, end-expiratory pressure for carbon dioxide; PETO_2_, end-expiratory pressure for oxygen; O_2_ sat, oxyhemoglobin saturation by pulse oximetry.*

**Figure 2:**
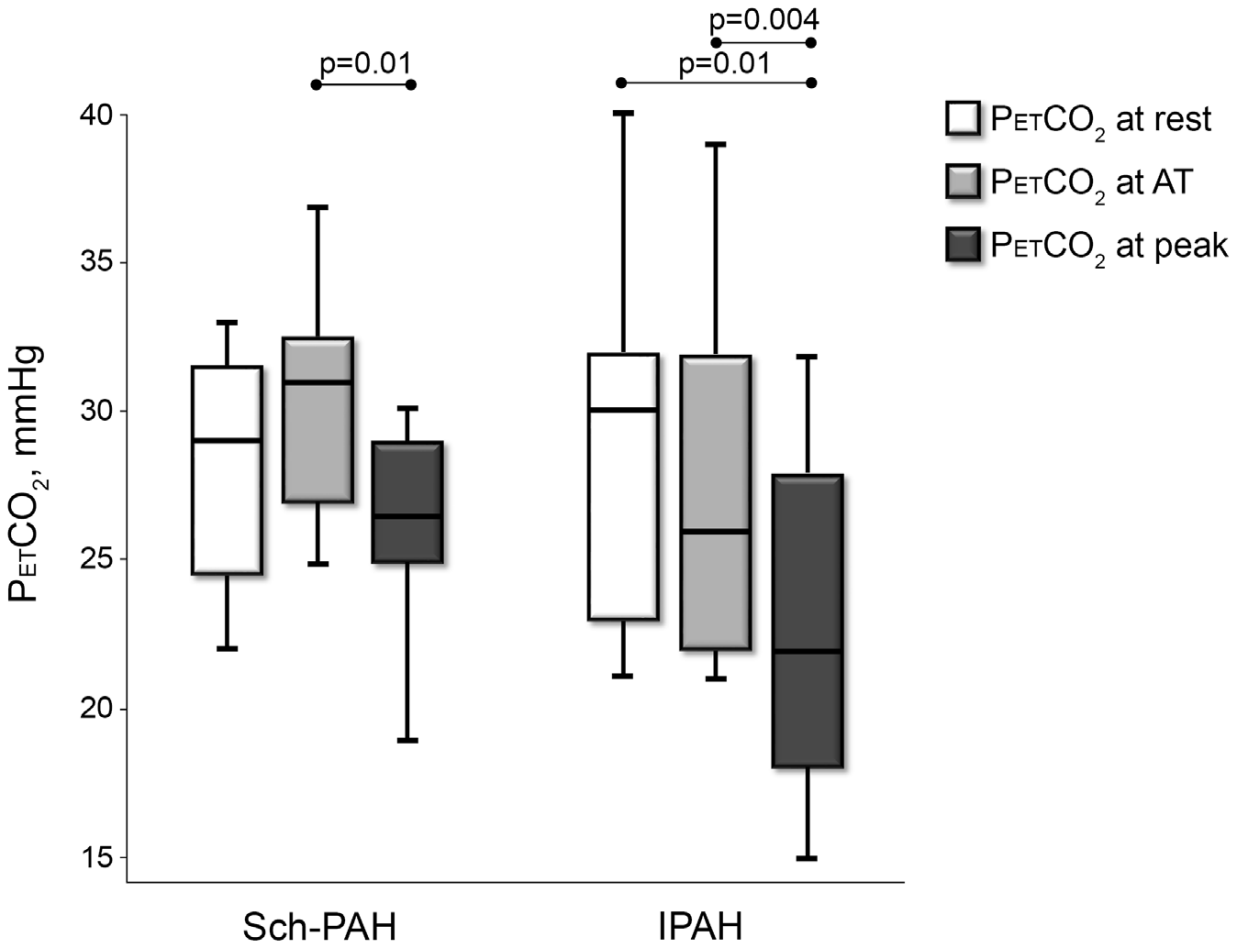
The behavior of PETCO_2_ between rest and maximal exercise in Sch-PAH and IPAH subjects.

Compared with IPAH patients, Sch-PAH subjects had greater peak VO_2_ and higher ΔVO_2_/ΔWR. Also, VO_2_ was higher in the Sch-PAH group at the AT. The ΔVE/ΔVCO_2_ was lower in Sch-PAH patients than IPAH patients. Although VE/VCO_2_ and PETCO_2_ at AT were smaller in Sch-PAH patients, these variables did not reach statistical significance. There was a trend for different PETCO_2_ behavior between the groups during exercise (Figure 2).

Sch-PAH patients had a shallower heart rate response for a given change in VO_2_ and, accordingly, a greater peak O_2_ pulse compared to IPAH subjects (p<0.05). No difference in SpO_2_ at rest and peak exercise was found between the groups. Mild hypoxemia during the test was only noted in 3 patients, 2 of which were from the Sch-PAH group.

Resting cardiac index was significantly correlated to peak VO_2_ only in IPAH subjects. Likewise, there was a correlation between peak VO_2_ and resting CI with VO_2_-WR and VE-VCO_2_ slopes in patients with IPAH, but not in subjects with Sch-PAH. In both groups, there were correlations between resting CI and peak VO_2_, with NYHA-FC, but not with 6MWT (Table 4; Table 5).

**Table 4:** Correlation coefficients between resting CI and key exercise variables obtained during CPET, NYHA-FC and 6MWT in patients with Sch-PAH and IPAH.

	Sch-PAH	IPAH
NYHA-FC	−0.85*	−0.74*
6MWD, m	−0.03	0.25
VO_2_ peak, % predicted	0.51	0.72*
ΔVO_2_/ΔWR	0.61	0.58*
ΔVE/ΔVCO_2_	0.08	−0.60*

* p<0.05;

*Definition of Abbreviations: NYHA-FC, New York Heart Association functional class; VO_2_, oxygen uptake; WR, work rate; VE, minute ventilation; VCO_2_, carbon dioxide uptake.*

**Table 5:** Correlation coefficients between VO_2_ peak (% predicted) with: NYHA-FC, 6MWT and selected CPET variables in patients with Sch-PAH and IPAH.

	Sch-PAH	IPAH
NYHA-FC	−0.82**	−0.83**
6MWT, m	−0.12	−0.05
ΔVO_2_/ΔWR	0.36	0.87**
ΔVE/ΔVCO_2_	−0.28	−0.95**

* p<0.05;

** p<0.01;

*Definition of Abbreviations: NYHA-FC, New York Heart Association functional class; VO_2_, oxygen uptake; WR, work rate; VE, minute ventilation; VCO_2_, carbon dioxide uptake.*

## Discussion

To the best of our knowledge, this is the first study to comparatively assess the physiological adjustments to progressive exercise in subjects with Sch-PAH and IPAH. Although it is an initial study with a small sample, our results indicated that Sch-PAH patients exhibit less impaired exercise responses than IPAH patients, even if they had similar resting pulmonary hemodynamic abnormalities. These data might provide a mechanistic explanation for the reported differences in disease progression.

There are potentially more than twice the number of people with Sch-PAH than IPAH worldwide. [2], [3] Even in non-endemic regions, the prevalence of schistosomiasis is relevant, as demonstrated in a recent report from the French National Center for PAH that showed that schistosomiasis was present in 11% of patients with PAH attributed to non-cirrhotic portal hypertension. [21] Little is known about the pathophysiology of Sch-PAH, and it was previously classified as PAH attributed to embolic phenomena – schistosoma eggs from the portal-hepatic system. [22], [23] However, later studies showed that the histopathological features of Sch-PAH were similar to IPAH, even with the absence of eggs in the pulmonary arteries. It is possible that the presence of schistosoma eggs in the portal veins could trigger an inflammatory response or an imbalance in the vasoactive mediators in the pulmonary arteries. [23].

Fernandes et al. [4] showed that Sch-PAH patients present with less severe disease, exhibiting more preserved resting pulmonary hemodynamics and a trend toward better functional status than IPAH patients. They reported similar survival rates between naïve Sch-PAH subjects and IPAH subjects treated with vasodilators, suggesting a more benign clinical course for Sch-PAH. The baseline characteristics of our patients in both groups were similar to those described by Fernandes et al. [4], and we did not find a trend toward better NYHA-FC, 6MWD and resting hemodynamics in Sch-PAH patients. We did, however, find striking differences between groups during the evaluation of some of the exercise responses obtained in the CPET, as described below.

### Differences in Aerobic Capacity and Work Efficiency

In healthy subjects, the VO_2_ increases as a function of cardiac output and O_2_ extraction, responding to muscle demand during exercise. In PAH, this response could mainly be impaired due to the failure of the right heart to increase cardiac output during exercise. Sun et al. [8] analyzed CPET findings in PAH patients with a mean cardiac index of 2.2 L/min/m^2^ and found a peak VO_2_ of about 44%, slightly lower than the values we found in our IPAH subjects. In our study, the peak VO_2_ was significantly higher in Sch-PAH patients compared to IPAH patients, even when expressed as mL/kg/min or a percent-predict value, which minimizes any heterogeneity between the groups. Furthermore, our subjects with IPAH had lower VO_2_ values at AT than our Sch-PAH patients, indicating the earlier development of lactic acidosis during exercise.

Moreover, the slope of VO_2_-WR, an index known to reflect circulatory or peripheral dysfunction, has been used to evaluate aerobic efficiency. This slope in IPAH patients was shallower than in Sch-PAH patients, reflecting a higher dependence on anaerobic metabolism during exercise in IPAH. [8], [19].

We did not find differences in 6MWD between the groups. Moreover, there were no correlations between 6MWD and parameters of aerobic capacity assessed by CPET, even with NYHA-FC. Although its importance in day practice, it is possible that the submaximal nature of the test, with lesser cardiovascular and ventilatory stress, had limited its capacity to detect disparities between the groups when compared to CPET. [24], [25].

### Differences in Ventilatory Efficiency and Gas Exchange

PAH patients often have a ventilatory inefficiency, which is defined as an abnormally high ventilatory response relative to CO_2_ output and expressed during CPET as either a ratio at the AT or a slope obtained below the AT. This response has been related to areas of high V/Q mismatching or increased alveolar ventilation associated with several mechanisms, and is proportional to PAH severity. [7], [8], [26] A VE-VCO_2_ slope greater than 48 is associated with limited survival in PAH [13]. In our study, this slope was above 48 in 55% of IPAH patients, however all Sch-PAH subjects had this slope bellow this level. Moreover, this slope was significantly steeper in IPAH subjects than in Sch-PAH subjects (Table 3; Figure 1). Severe arterial hypoxemia and the development of right-to-left shunting during exercise could be additional explanations for the increases in ΔVE/ΔVCO_2_ [27] but they were not present in our patients.

Excessive ventilation in PAH patients is often accompanied by a reduction in PETCO_2_. In healthy subjects, a rise in PETCO_2_ is expected between rest and the AT, with a subsequent reduction in PETCO_2_ at maximal exercise. [8], [28] In our study, there was a trend toward lower PETCO_2_ values at AT in IPAH patients. Moreover, there was a tendency for PETCO_2_ to rise between rest and AT in Sch-PAH patients, contrasting with a progressive fall in PETCO_2_ in IPAH subjects (Figure 2), suggesting more preserved gas exchange function in Sch-PAH. Hansen et al. [29] emphasized the exercise-induced decline in PETCO_2_ as a marker for PAH as opposed to COPD and left ventricular failure. Furthermore, Yasunobu et al. [28] showed that the exercise-induced fall in PETCO_2_ that is observed in PAH is proportional to the severity of the disease.

### Differences in Cardiovascular Responses to Exercise

O_2_ pulse (VO_2_/HR) is determined by stroke volume and O_2_ extraction. In our study, as there was an absence of severe oxyhemoglobin desaturation, the higher peak O_2_ pulse seen in Sch-PAH subjects probably reflects greater increases in stroke volume during exercise compared to IPAH subjects. Likewise, the inability to augment stroke volume during exercise leads to a disproportional increase in heart rate as a function of the metabolic demand (ΔHR/ΔVO_2_). This response was predominantly observed in our patients with IPAH, as opposed to Sch-PAH (Table 3; Figure 2).

### Correlations between Resting Hemodynamics and Exercise Measurements

The resting cardiac index is known to correlate well with functional limitation in IPAH. [10]. Accordingly, in our patients, a correlation of resting CI to peak VO_2_ was observed in IPAH subjects, but not in Sch-PAH. Also, we found a significant correlation between peak VO_2_ and resting CI with ventilatory (ΔVE/ΔVCO_2_) and aerobic efficiency (ΔVO_2_/ΔWR) only in IPAH patients (Tables 4 and 5). Although no definite conclusions can be made on the correlation values, this is additional evidence of a more pronounced cardiovascular limitation in IPAH, indicating that patients with Sch-PAH have a greater ability to increase pulmonary blood flow during exercise, even with similar resting pulmonary hemodynamics (Table 2). Moreover, these finding supports the contention that selected CPET variables should be used with caution when assessing the prognosis or severity of patients with non-idiopathic PAH, as stated in a recent study involving various etiologies of PAH. [30].

### Study Limitations

There are some limitations in our study that need to be acknowledged. The single-center nature of the study and our location in an area of Brazil that is not endemic for schistosomiasis may account for some selection bias; however, this bias is minimized by intern migratory practices to a cosmopolitan city like São Paulo. Also, as a result of restricted selection criteria, this study included a small number of patients, which limit the extrapolation of the results for different phenotypes of Sch-PAH subjects; moreover, the probability of a type II error must be considered in some comparisons between the groups. The female preponderance in IPAH group might explain some differences observed during CPET; however, the responses in percent-predict (gender-adjusted) were consistent with those found in absolute values, which minimizes this heterogeneity. Also, we were unable to evaluate arterial blood gases in all subjects during exercise, making it impossible to accurately assess the behavior of VD/VT.

In conclusion, patients with Sch-PAH had higher aerobic capacity, lower submaximal ventilatory and cardiovascular stresses, and less impaired pulmonary gas exchange abnormalities than IPAH subjects with similar resting hemodynamic dysfunction. These findings suggest that the more benign clinical course of Sch-PAH could be due to the less impaired ability of the right ventricle to increase pulmonary blood flow during exercise, as compared to IPAH subjects. This hypothesis deserves further investigation in larger epidemiological studies.
